# Acknowledgement to Reviewers of *Medicines* in 2017

**DOI:** 10.3390/medicines5010015

**Published:** 2018-01-29

**Authors:** Medicines Editorial Office

**Affiliations:** MDPI AG, St. Alban-Anlage 66, 4052 Basel, Switzerland

Peer review is an essential part in the publication process, ensuring that *Medicines* maintains high quality standards for its published papers. In 2017, a total of 87 papers were published in the journal. Thanks to the cooperation of our reviewers, the median time to first decision was 17 days and the median time to publication was 47 days. The editors would like to express their sincere gratitude to the following reviewers for their time and dedication in 2017:
Abraham, John PLee, Jun-hwanAdams, JamesLee, Meng-shiouAkiba, EtsukoLi, ChunshunAl-Fatimi, MohamedLi, DapengAmant, CaroleLi, ShaoAmorati, RiccardoLiakos, Ioannis L.Angiolella, LetiziaLiao, Jyh-feiAnne, JozefLin, Ming-KuemAnraku, MakotoLo Scalzo, RobertoAssenza, GiovanniLognay, GeorgesAurora, RajeevLoh, ZhixuanAverbeck, DietrichLopez Rodilla, Jesus M.Backes, ToddLu, Mei-ChinBamford, ConnorMa, Sheng-xingBenzeroual, Kenza E.Mabou Tagne, AlexBeretta, GiangiacomoMalde, AlpeshBian, ZhaoxiangManfredini, StefanoBianco, I.D.Manjunath, ManuboluBonaiuto, VincenzoManninger, MartinBosch, M.P.C.Manolopoulos, SpyrosBožović, MijatMartins, Maria RosarioBuonanno, ManuelaMartins, NatáliaButler, MarkMatsumoto, YasuhikoCabral, CéliaMayhan, William G.Cádiz Gurrea, María De La LuzMazzoni, LucaCai, LishengMiccadei, StefaniaCampana, RaffaellaMichiels, JorisCampbell, FionaMiguel, GraçaCanini, AntonellaMinelli, AlbaCapasso, RaffaeleMist, Scott D.Carrubba, AlessandraMitakou, SofiaCasalino, ElisabettaMnif, WissemChae, Youn ByoungMok, Daniel Kam-WahChang, Long-SenMollica, AdrianoChao, Pi-YuMorel-Rouhier, MélanieChazot, Paul LMorikawa, ToshioCheriyath, VenugopalanMosawy, SaphaChiessi, EsterMulas, MaurizioChirumbolo, SalvatoreNaito, YujiChizzola, RemigiusNandha Premnath, PadmavathyChou, Cheng-YangNaranjo-Hernández, DavidChristova-Bagdassarian, ValentinaNobili, StefaniaChu, ShidongObata, YasukoÇiçek, Serhat SezaiOh, ByeongsangCleary, Margot P.Pacurari, MaricicaCoelho, José APalla, FrancoCosta, RosariaPalomino, Olga MaríaCulliford, LarryPapetti, AdeleDai, YuminPappa, AglaiaDalessandro, GiuseppePark, Il-KwonDall'Acqua, StefanoPathak, RupakDas, UndurtiPaulsen, Berit SmestadDe Falco, EnricaPayne, PeterDe Gracia, PabloPereira, TâniaDel Bo, CristianPerepelyuk, MarynaDi Stefano, AntonioPeterson, JuliaDwivedi, ChandradharPfragner, RoswithaEedunuri, Vijay KumarPiriou, YannickEggenhöffner, RobertoPirzada, TahiraElsinghorst, Paul W.Plano, DanielEspuelas, SocorroPorter, DiannaFaralli, AdeleProestos, CharalamposFerreira, Rui M.Qu, XianqinFlamini, GuidoQuinn, Mark T.Flower, AndrewRambold, Holger A.Foti, Mario C.Ramsey, Vincent K.Franz, ChlodwigRauch, BernhardFrosini, MariaReis, FlávioFurneri, Pio MariaRigano, DanielaGao, Xin-YanRimawi, Fuad AlGazzaniga, V.Rivero-Pérez, M. DoloresGhatak, ArindamRoby, Mohamed Hussein HamdyGiamperi, LauraRubiolo, Pat6riziaGiarratana, FilippoRusso, MarinaGonzález De Llano, DoloresSaha, AchintoGrace-Farfaglia, PatriciaSaiano, FilippoGreen, BenSakamoto, ToshioGross, AaronSansone, AnnaGrosso, ClaraSatchidanand, NikhilGuo, LeiSatyal, PrabodhGverzdys, TomasSawynok, JanaHammerl, Jens AndreSchettino, GiuseppeHano, ChristopheScudiero, OlgaHatano, TsutomuSegura, JuanHenniges, UteSerafini, GianlucaHewlings, Susan J.Serra, StefanoHo-Cheol, KimSetzer, William N.Hoefer, JuliaShankar, EswarHolzel, ChristinaShieh, Tzong-MingHook, IngridShirai, YoshihiroHosokawa, SeijiroSillup, George P.Houbracken, IsabelleSilva, Gabriela JorgeHsu, TcSingh, Atul KumarHu, JimSmeds, Annika I.Hu, MingKuanSoulika, AthenaHwang, In KooStanley, JoneIbrahim, SalamSugimoto, SachikoIrusta, SilviaSukocheva, OlgaIvankin, AndreyTanaka, Tim HideakiJaaro-Peled, HannaTedesco, IdoloJaradat, NidalTomi, FélixJayant, Rahul DevTripodi, GianlucaJeong, Seon-YongTullio, VivianJiang, XuezhiTzang, Bor-ShowJin, ZhaoUmehara, KaoruJirovetz, LeopoldUsai, MariannaJuliano, ClaudiaValko, Philipp O.Kamdem, PascalValomon, AmandineKao, Ming-ChingVan Den Noort, Maurits W.M.L.Kathuria, HimanshuVarikuti, SanjayKhalil, ChristianViñas, MiguelKim, Dong JoonVitalini, SaraKim, No SooVora, MehulKim, SoJungWanchai, AusaneeKlein, PenelopeWang, Chin-KunKluza, JéromeWang, MeiKnupp, GerdWang, ZhijunKojima-Yuasa, AkikoWeiner, Shira SchecterKomaki, HisayukiWertz, Philip W.Kong, Chang-SukWilkinson, J. M.Kontogiorgis, ChristosWilson, DouglasKouretas, DimitriosWu, Shu-JuKoziel, JacekWu, XinyinKuang, HuihuiYang, LinKubota, NaotoYang, Wei-hsiungKwok, TimothyYang, ZhengyuLacaille-Dubois, Marie-AlethYu, Yong-mingLayek, BuddhadevZehr, PaulLeclercq, GuyZhang, FanLee, Dong HunZi, XiaolinLee, GihyunZygadlo, Julio

